# In Vitro and In Vivo Efficacy of a Novel Glucose–Methotrexate Conjugate in Targeted Cancer Treatment

**DOI:** 10.3390/ijms22041748

**Published:** 2021-02-09

**Authors:** Marta Woźniak, Gabriela Pastuch-Gawołek, Sebastian Makuch, Jerzy Wiśniewski, Tibor Krenács, Peter Hamar, Andrzej Gamian, Wiesław Szeja, Danuta Szkudlarek, Monika Krawczyk, Siddarth Agrawal

**Affiliations:** 1 Department of Pathology, Wroclaw Medical University, 50-367 Wroclaw, Poland; marta.wozniak@umed.wroc.pl (M.W.); sebastian.makuch@student.umed.wroc.pl (S.M.); danuta.szkudlarek@umed.wroc.pl (D.S.); 2 Department of Organic Chemistry, Bioorganic Chemistry and Biotechnology, Faculty of Chemistry, 44-100 Gliwice, Poland; gabriela.pastuch-gawolek@polsl.pl (G.P.-G.); wieslaw.szeja@polsl.pl (W.S.); 3 Biotechnology Centre, Silesian University of Technology, 44-100 Gliwice, Poland; 4 Department of Medical Biochemistry, Wroclaw Medical University, 50-367 Wroclaw, Poland; jerzy.wisniewski@hirszweld.pl; 5 Department of Immunology of Infectious Diseases, Hirszfeld Institute of Immunology and Experimental Therapy, Polish Academy of Sciences, 53-114 Wroclaw, Poland; andrzej.gamian@hirszfeld.pl; 6 Department of Pathology and Experimental Cancer Research, Semmelweis University, 1085 Budapest, Hungary; krenacst@gmail.com; 7 Institute of Translational Medicine, Semmelweis University, 1085 Budapest, Hungary; hamar.peter@med.semmelweis-univ.hu; 8 Department and Clinic of Internal Medicine, Occupational Diseases, Hypertension and Clinical Oncology, Wroclaw Medical University, 50-367 Wroclaw, Poland

**Keywords:** glycoconjugates, methotrexate, cancer treatment, glucose metabolism, drug design and discovery, anticancer drugs, targeted therapy, Warburg effect

## Abstract

Methotrexate (MTX) is a commonly used antimetabolite, which inhibits folate and DNA synthesis to be effective in the treatment of various malignancies. However, MTX therapy is hindered by the lack of target tumor selectivity. We have designed, synthesized and evaluated a novel glucose–methotrexate conjugate (GLU–MTX) both in vitro and in vivo, in which a cleavable linkage allows intracellular MTX release after selective uptake through glucose transporter−1 (GLUT1). GLU–MTX inhibited the growth of colorectal (DLD-1), breast (MCF-7) and lung (A427) adenocarcinomas, squamous cell carcinoma (SCC-25), osteosarcoma (MG63) cell lines, but not in WI-38 healthy fibroblasts. In tumor cells, GLU–MTX uptake increased 17-fold compared to unconjugated MTX. 4,6-O-ethylidene-α-D-glucose (EDG), a GLUT1 inhibitor, significantly interfered with GLU–MTX induced growth inhibition, suggesting a glucose-mediated drug uptake. Glu-MTX also caused significant tumor growth delay in vivo in breast cancer-bearing mice. These results show that our GLUT-MTX conjugate can be selectively uptake by a range of tumor cells to cause their significant growth inhibition in vitro, which was also confirmed in a breast cancer model in vivo. GLUT1 inhibitor EDG interfered with these effects verifying the selective drug uptake. Accordingly, GLU–MTX offers a considerable tumor selectivity and may offer cancer growth inhibition at reduced toxicity.

## 1. Introduction

Methotrexate (MTX) is among the most widely applied and effective therapeutic agents available to treat various cancers, including breast cancer, lung cancer, bladder carcinoma, and osteogenic sarcoma, as well as autoimmune diseases [1]. However, MTX has a number of deficiencies that arise from a lack of tumor selectivity [2,3]. The pharmacokinetic parameters of MTX are unsatisfactory and frequently result in an insufficient clinical response. Increasing the dose of MTX may result in higher therapeutic efficacy, but it also leads to a greater risk of side effects [4]. In general, the principal reason for the discontinuation of MTX is not the lack of efficacy but life-threatening toxicity. We addressed these limitations by designing a next-generation tumor-targeting MTX delivery system for improved safety and efficacy.

One of the attractive strategies to achieve the desired specificity is to connect a therapeutic agent with a ligand that selectively interacts with the pathological cell. To increase the safety and efficacy of the therapy, the synthesis of a prodrug in which the chemotherapeutic is bound to a ligand with a high-affinity for diseased cells is required. To achieve this, the diseased cells must overexpress the ligand-specific receptor that could facilitate the targeted uptake of the therapeutic agent. Examples of such prodrugs include peptide-drug, antibody–drug, aptamer-drug, and folic acid–drug conjugates [5].

In order to sustain the growth and proliferation, malignant cells significantly increase glucose uptake and the flux of substrates through glycolysis even under oxidative conditions. This abnormality, termed “the Warburg effect,” originates from mitochondrial metabolic changes and is one of cancer’s most common traits [6]. The elevated glucose intake requires the overexpression of glucose transporters (GLUTs), which is frequent in neo-plasms and provide clinical targets for therapy [7,8]. Therefore, glycoconjugation, in which cytotoxic agents or targeted anticancer therapeutics have been linked to glucose, can improve the selective uptake of anticancer drugs [9,10,11,12,13,14]. Since the introduction of glufosfamide [15], the potential of this strategy in diagnosis and therapy has already been realized, yet there is tremendous scope for improvement [7].

**Figure 1:**
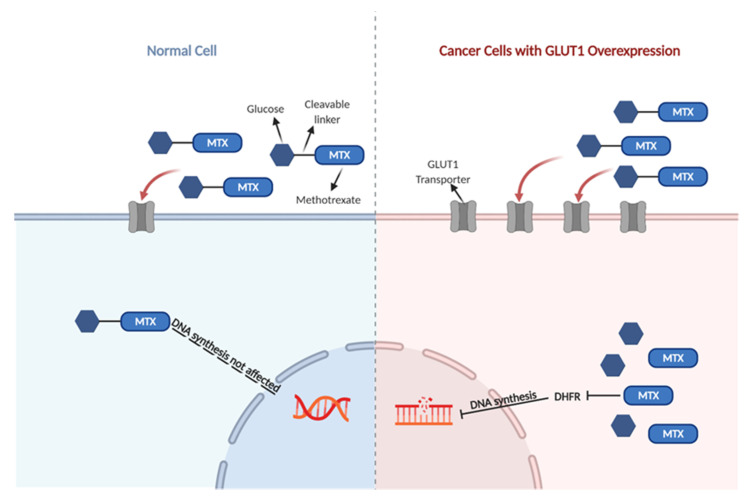
Cellular transport of glucose–methotrexate conjugate (GLU–MTX) in healthy and cancer cells with GLUT1 overexpression. The intracellular cleavage of acid-labile bonds in the acidic environment of cancer cells results in controlled release of MTX, which inhibits dihydrofolate reductase (DHFR) and leads to cell death. In healthy cells, no major effect in DNA synthesis is observed.

These ligand-targeting drugs (LTD) are constructed by conjugating a cleavable linker to the payload. The efficacy of such conjugate is primarily determined by the therapeutic agent activity, while the safety of the conjugate is dictated by the ligand specificity on the tumor cell. Separating the diseased cell selectivity and therapeutic drug activity is the most critical step which can be independently optimized. Therefore, we first focused on determining the optimal structure of the glucoside, through which the sugar is locked to the linker, then the transfer of the conjugate to tumor cells and its uptake mediated by GLUT1 protein. Based on these considerations, we designed, synthesized, and biologically evaluated a novel glucose–methotrexate conjugate (GLU–MTX), in which MTX, D-Glucose, and the linker are connected via a cleavable linkage susceptible to the action of hydrolytic enzymes. To the best of our knowledge, no one has previously synthesized or evaluated the conjugation of glucose to MTX. In our recent study, we showed that GLU–MTX exerts a strong cytotoxic effect on breast and colon cancer cells and displays an increased selectivity in the tumor microenvironment [16]. These findings conclusively prove the potential of glycoconjugation for the selective destruction of cancer cells by MTX. In view of this, the objective of this study was to further evaluate the efficacy of GLU–MTX on a wide variety of cancer cell lines as well as in vivo and examine the mechanisms underlying the cellular transport of glucose–methotrexate conjugate. The present study revealed that GLU–MTX is a potent therapeutic agent that preferentially accumulates in and annihilates cancer cells at reduced toxicity in the noncancerous tissues (Figure 1).

## 2. Results

### 2.1. Synthesis of Sugar Derivative Emerging from GLU–MTX Conjugate Hydrolysis in Tumor Cells

**Scheme 1:**
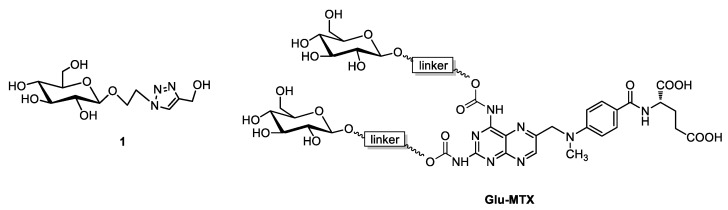
Structures of the glucoconjugate **1** and GLU–MTX glycoconjugate.

The initial stage of the research was the synthesis of a glucoconjugate **1** containing a D-glucose- unit linked via a glycosidic bond with a linker (Scheme 1). The conjugate 1 was prepared by the 1,3-dipolar cycloaddition reaction of 2-azidoethyl β-D-*O*-glucopyranoside and propargyl alcohol. As indicated in the experimental section, the results suggest that the uptake of the glucoconjugate **1** is mediated by the GLUT1 transporter. During these studies, it was also noticed that this compound has a weak cytotoxic effect. Based on the encouraging result that glucoconjugate **1** was transferred into the tumor cells, we synthesized prodrug GLU–MTX in the cycloaddition reaction according to the method developed by Sharpless. The substrates for this reaction were 2-azidoethyl β-D-*O*-glucopyranoside and MTX di-propargylcarbamide derivatives, obtained by reacting the propargyl chloroformate with an antibiotic (MTX) in the presence of *N*-methylimidazole (NMI) and tertiary amine such as *N*,*N*-diisopropylethylamine in methylene chloride as a solvent [16].

GLU–MTX exhibits comparable antiproliferative activity to MTX against different cancer cell lines and has higher selectivity for cancer cells over normal cells in vitro.

**Figure 2:**
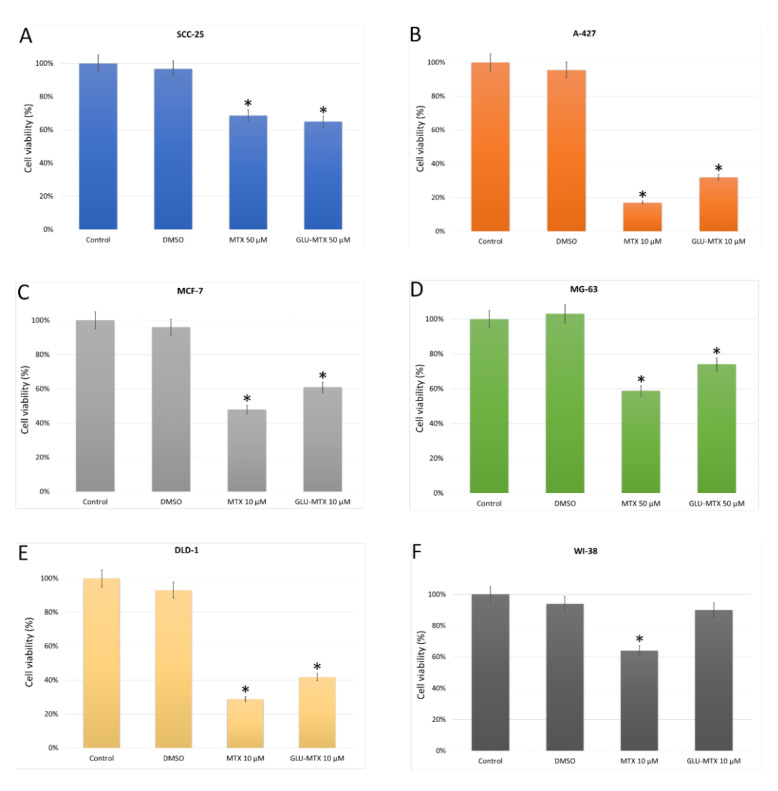
Cell viability of various human cancer cell lines: squamous cell carcinoma SCC-25, lung carcinoma A-427, human colon adenocarcinoma DLD-1, breast carcinoma MCF-7, osteosarcoma MG-63 (**A**–**E**) and normal human fibroblast WI-38 (**F**) after MTX and GLU–MTX treatment for 48 h at doses 10–50 μM. Results are presented as means ± standard deviations from three independent experiments. * *p* < 0.05 vs. control.

GLU–MTX and MTX were first tested for their in vitro cytotoxicity with the use of the MTT assay. Six cell lines representing five types of human malignancies (breast, colon, skin, lung, bone) were cultured with test compounds at concentrations in the range of 10 to 50 μM for 48 h; then, cell viability was determined. The findings demonstrated that GLU–MTX had a similar cytotoxic effect compared to MTX on the SCC-25 skin cancer cell line (Figure 2A). Cell viability of the other MTX-treated cell lines was slightly lower (16–19% depending on the cell line) (Figure 2B–E than the viability of the same dose of GLU–MTX-treated cells. We compared the selectivity of GLU–MTX and MTX using various cancer cell lines to match the results with a healthy fibroblast WI38 cell line. As shown in Figure 2F, we found that the cellular viability of WI-38 was significantly higher in GLU–MTX-treated cells compared to MTX-treated cells. This result indicates that GLU–MTX is less cytotoxic to healthy cells than MTX.

### 2.2. The Cytotoxic Effect of GLU–MTX Is Reversed by GLUT1 Inhibitor

**Figure 3:**
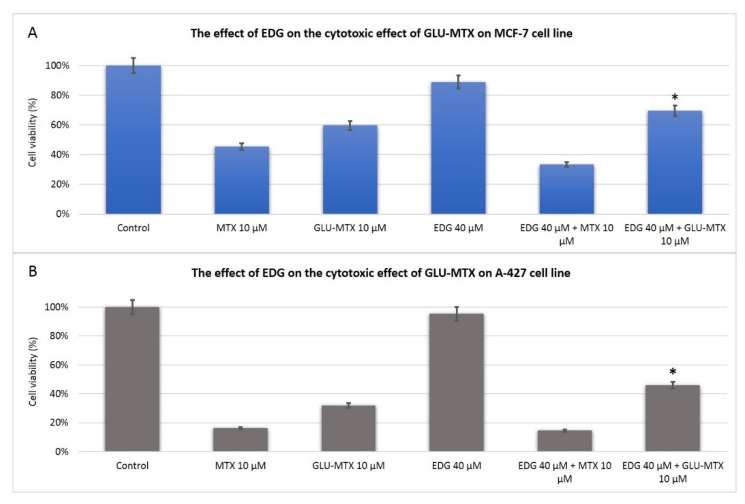
The effect of glucose transporter−1 (GLUT1) inhibitor 4,6-O-ethylidene-α-D-glucose (EDG) on the efficacy of free MTX and GLU–MTX treatment of (**A**) breast cancer cell line MCF-7 and (**B**) lung cancer cell line A-427. Cells were preincubated with EDG for 4 h and then incubated for 48 h with MTX or GLU–MTX. Results are presented as means ± standard deviations from two independent experiments. * *p* < 0.05 vs. GLU–MTX 10 μM.

The cytotoxicity assay was carried out in the absence and presence of an exofacial GLUT1 competitive inhibitor 4,6-O-ethylidene-α-D-glucose (EDG). Cells preincubated with EDG and then with conjugated MTX had a lower cell death ratio in comparison to cells incubated with free MTX in both cell lines. MCF-7 and A-427 cells viability after EDG + MTX treatment was 35% and 15%, respectively, whereas following EDG + GLU–MTX was 70% and 50%, respectively (Figure 3). The MTT assay results support the hypothesis that glucose transporter GLUT1 is involved in the cellular uptake of glucose conjugate MTX.

### 2.3. Cellular Uptake of GLU–MTX Is Significantly Higher in SW-480 Colon Cancer Cells Compared to Free MTX

**Figure 4:**
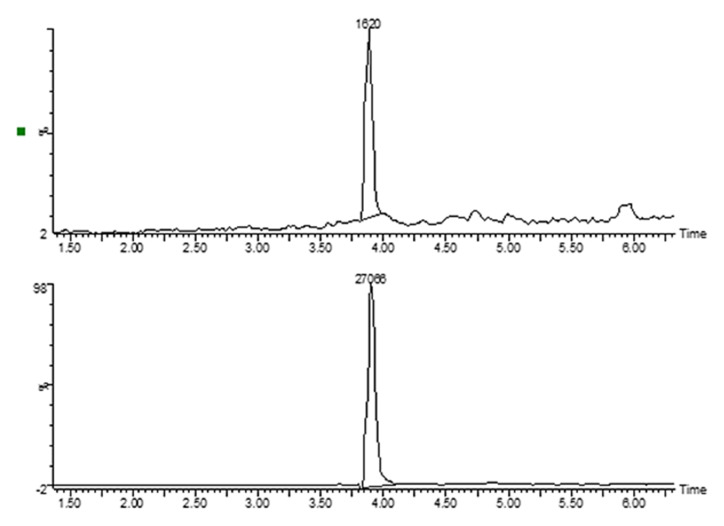
Mass chromatograms for m/z 455.18 ions in extracts from colon cancer SW-480 cell line—upper chromatogram MTX, lower GLU–MTX.

GLU–MTX is transported by facilitated diffusion exploiting overexpressed GLUT1 transporters [17] and is approximately 17-times more preferentially accumulated in cancer cells compared to free MTX (Figure 4). In the intracellular compartment, the cleavage of acid-labile bonds occurs, which results in the controlled release of free MTX.

### 2.4. Both MTX and GLU–MTX Lead to Cell Cycle Arrest in S Phase

**Figure 5:**
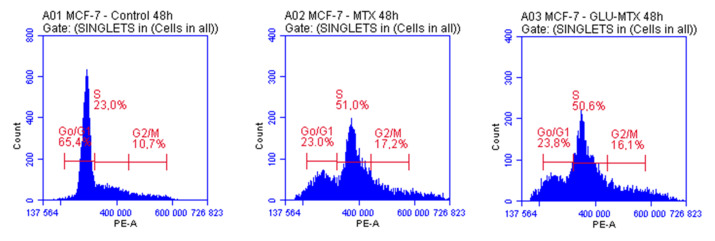
The effect of MTX and GLU–MTX on MFC-7 cell cycle progression.

To investigate whether MTX and GLU–MTX display the same mode of action, their effect on cell cycle progression was examined on the MCF-7 cell line. The results showed that cell populations in the S phase were significantly higher in MTX and GLU–MTX-treated cells after 24 h, in contradistinction with untreated cells (Figure 5, Table 1). This result proves that both compounds affect the cell cycle in a similar way, indicating the presence of free MTX originated from GLU–MTX in the intracellular compartment.

### 2.5. In Vivo Efficacy of GLU–MTX

**Figure 6:**
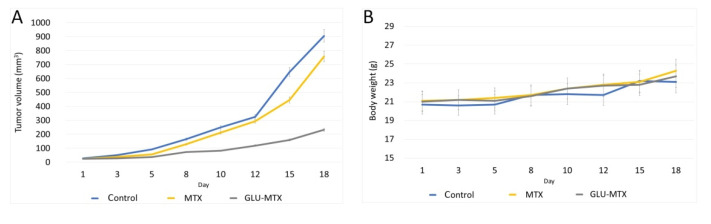
Results of the in vivo study. (**A**) The average tumor volume after 18 days in 3 groups (1—control, 2—MTX-treated and 3—GLU–MTX treated). In group 3, the tumor volume was significantly lower compared to other groups, as analyzed by one-way ANOVA followed by Bartlett’s test (*p* < 0.001). (**B**) The effect of the therapy on body weight. No significant loss of body weight has been observed.

After our observations of its potent in vitro effects, GLU–MTX and MTX were evaluated on 4T1 breast tumor-bearing mice, which are characterized by GLUT1 overexpression [18]. The compounds were injected i.v. in a single dose (day 0). MTX was given at 120 mg/kg, and GLU–MTX was given at a corresponding dose of 300 mg/kg. GLU–MTX significantly inhibited 4T1 allograft tumor growth by about 74.4% on day 18 (*p* < 0.01), whereas MTX led to tumor growth inhibition by 16.2% (Figure 6A). There was no significant loss of body weight in neither of the treatment groups (Figure 6B).

**Figure 7:**
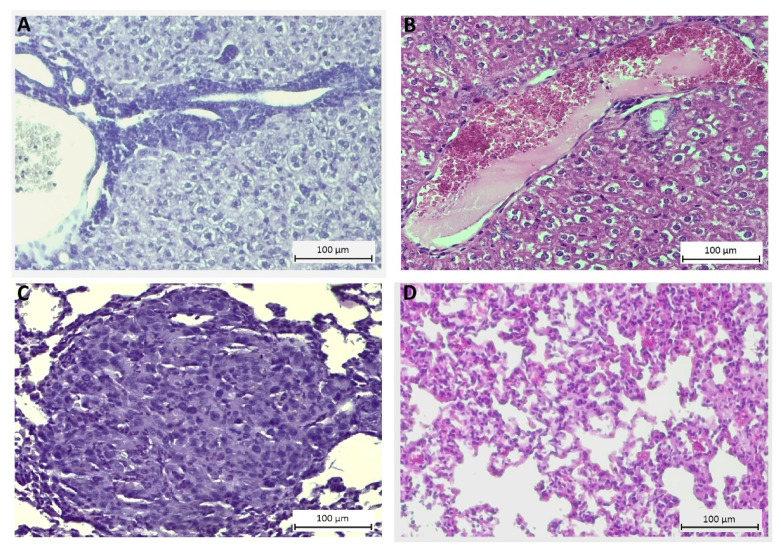
Representative images of liver and lung sections stained with hematoxylin–eosin (HE) to evaluate the cytotoxic impact of the MTX and GLU–MTX on the mice tissues. (**A**) Liver with typical for MTX therapy periportal inflammation. (**B**) The liver without pathological changes after GLU–MTX treatment. (**C**) The lung section shows lymphocytic and plasmacytic infiltration in mice treated with MTX. (**D**) Lung section without significant changes in the morphology from mice after treatment of GLU–MTX. Optical magnification: 200×. Scale bars 100 μm.

Histopathological analyses of the liver and lungs excised from MTX and GLU–MTX-treated mice showed differences in tissue morphology (Figure 7). Liver sections after MTX-treatment indicate visible periportal inflammation, while lung sections show lymphocytic and plasmacytic infiltration. Livers and lungs from GLU–MTX treated mice were without any morphological changes. Thus, the results indicate that GLU–MTX significantly inhibited tumor growth without affection of livers and lungs compared to free MTX.

## 3. Discussion

The “Warburg effect,” the increased aerobic glycolysis in many malignancies, has been extensively scrutinized and is now suggested to be the reason for most of the hallmarks of cancer [19,20]. Metabolic differences between normal and cancer provide an environment that often results in drug resistance. However, these characteristic features may also provide an opportunity to design appropriately tailored molecular targeted oncotherapy interventions.

This study represents the first attempt to systematically evaluate the anticancer activities of a novel glucose–methotrexate conjugate in vitro and in vivo. We have shown several essential characteristics of this drug: (a) GLU–MTX exhibits potent anticancer activity against a range of solid tumor cell lines with IC50 values similar to free MTX; (b) GLU–MTX preferentially annihilates cancer cells while showing low toxicity in noncancerous cells in vitro; (c) cellular uptake of GLU–MTX is glucose-transporter-specific; (d) the uptake of GLU–MTX in cancer cells is 17 times more efficient than that of MTX; (e) Glu-MTX caused significant tumor growth delay in breast tumor-bearing mice compared to MTX-treated and control mice.

Our results indicate that GLU–MTX may be used against a broad spectrum of cancers. Glu- MTX cytotoxicity consistently had IC50 values in the µmol/L range. The compound exerted higher selectivity for cancer cells over normal cells. The translocation efficiency and subsequent cellular accumulation were significantly higher in GLU–MTX-treated cells than in MTX. Notably, our results indicate that glucose transporter GLUT1 is involved in the cellular uptake of glucose conjugates. The viability of MTX-treated cells did not change significantly in the presence of an exofacial GLUT1 inhibitor. However, the GLUT1 inhibitor decreased the activity of GLU–MTX, which suggests that the reduced uptake of the compound resulted in lower cellular accumulation and weaker anticancer action. We cannot univocally state that the cellular transport of glucose conjugate is facilitated solely via the GLUT1 transporter. However, knowing that the glycoconjugates are highly hydrophilic, it is rather unlikely that their transport occurs via passive diffusion. Evidence has been found that the cellular uptake of some glycoconjugates may also be mediated by other receptors such as OCT2, SGLT, SWEET, and asialoglycoprotein receptor (ASGPR) [21,22]. These findings are corroborated by other studies showing that glucose conjugates exploit glucose transporters of cancer cells [9,17]. The uptake analysis showed that in the intracellular compartment, the payload was quickly detached from the conjugate. This suggests that the cleavable linkage allows the release of the cytotoxic payload inside the malignant cells, possibly through enzymatic hydrolysis. This finding is particularly significant as the spacer arm must be designed in such a way as to ensure its stability in the extracellular compartment while also allowing the action of the active cytotoxic payload addressed to tumor cells. The nature of the spacer thus influences how favorable drug delivery is and its outcome. Over-stable linkers can curb the activity of the associated pharmacophore, resulting in a low-potency compound. Conversely, an understable spacer can provoke poor target specificity and high systemic toxicity [23].

Our study bears several limitations. First, the synthesis is multi-staged and requires more delicate control of the experimental parameters. Hence, a limited amount of the compound was obtained for biological assays. Second, we were able to perform in vitro and in vivo analysis only on selected cancer cell models; hence the results may not be generalizable. Third, the in vivo study did not include multiple administrations of the tested compounds. Further analyses are required to examine these effects on an animal model.

## 4. Materials and Methods

### 4.1. Chemistry

NMR spectra were recorded with an Agilent spectrometer 400 MHz using TMS as internal standard and CDCl_3_ or DMSO-d6 as a solvent. NMR solvents were purchased from ACROS Organics (Geel, Belgium). Chemical shifts (δ) were expressed in ppm and coupling constants (J) in Hz. Optical rotations were measured with a JASCO *P*-2000 polarimeter using a sodium lamp (589.3 nm) at room temperature. Melting point measurements were performed on a Stanford Research Systems OptiMelt (MPA 100). Electrospray ionization mass spectrometry was performed on the Xevo G2 Q-TOF mass spectrometer. Reactions were monitored by TLC on precoated plates of silica gel 60 F254 (Merck Millipore, Burlington, MA, USA). The TLC plates were inspected under UV light (λ = 254 nm) or charring after spraying with 10% sulfuric acid in ethanol. Crude products were purified using column chromatography performed on silica gel 60 (70–230 mesh, Fluka, St. Louis, MI, USA) developed with toluene/EtOAc and CHCl_3_/MeOH as solvent systems. Organic solvents were evaporated on a rotary evaporator under diminished pressure at 40 °C. All of the chemicals used in the experiments were purchased from Sig-ma-Aldrich (Saint Louis, Missouri, USA), ACROS Organics (Geel, Belgium), and Avantor Performance Materials Poland S.A (Gliwice, Poland) and were used without purification. Methotrexate, propargyl chloroformate, propargyl alcohol, and D-glucose are commercially available. 2-Azidoethyl β-D-O-glucopyranoside [24,25] and GLU–MTX [16] was prepared according to the respective published procedures.

#### Synthesis of Glycoconjugate

2-Azidoethyl β-D-O-glucopyranoside (82 mg, 0.33 mmol) and propargyl alcohol (20 µL, 0.33 mmol) were dissolved in a dry solvent system: THF (3 mL) and *i*-PrOH (3 mL). The solutions of sodium ascorbate (27 mg, 0.13 mmol) in H_2_O (1.5 mL) and CuSO_4_·5H2O (16 mg, 0.06 mmol) in H_2_O (1.5 mL), mixed and immediately added to the reaction mixture. The reaction mixture was stirred for 24 h at room temperature. Then, the solvents were evaporated in vacuo, and the crude products were purified by column chromatography (dry loading: CHCl_3_:MeOH, gradient: 50:1 to 2:1) to give products **1** (69 mg, 70% yield): m.p. 60–63 °C; [α]_D_^22^= −5 (c = 1.0, DMSO).

^1^H NMR (400 MHz, DMSO-d6): δ 2.97 (m, 1H, H-2_Glu_), 3.04 (m, 1H, H-4_Glu_), 3.09–3.16 (m, 2H, H-3_Glu_, H-5_Glu_), 3.43 (m, 1H, H-6a_Glu_), 3.68 (m, 1H, H-6bGlu), 3.89 (m, 1H, CH), 4.07 (m, 1H, CH), 4.23 (d, 1H, J = 7.8 Hz, H-1_Glu_), 4.47–4.58 (m, 5H, 2xCH2, OH), 4.91 (d, 1H, J = 5.1 Hz, OH), 4.95 (d, 1H, J = 4.7 Hz, OH), 5.06 (d, 1H, J = 5.1 Hz, OH), 5.15 (dd, 1H, J = 5.5 Hz, J = 5.9 Hz, OH), 8.01 (s, 1H, H-5_triaz_).

13C NMR (100 MHz, DMSO-d6): δ 49.54, 54.99, 61.05, 67.41, 69.99, 73.29, 76.60, 76.97, 102.93, 123.45, 147.68.

HRMS (ESI-TOF): calcd for C_11_H_20_N_3_O_7_ ([M + H]^+^): m/z 306.1301; found m/z 306.1300.

### 4.2. Cell Culture

The panel of different human cell lines was used to evaluate the effectiveness of the novel compound, human colon adenocarcinoma SW-480 and DLD-1, breast carcinoma MCF-7, squamous cell carcinoma SCC-25 purchased from the Leibniz Institute DSMZ-German Collection of Microorganisms and Cell Cultures, (DSMZ, Braunschweig, Germany), lung carcinoma A427, osteosarcoma MG63, normal fibroblasts WI-38 kindly provided by Institute of Immunology and Experimental Therapy, the Polish Academy of Sciences, Poland, and obtained from American Type Culture Collection. Cell lines A427, MG63, WI-38 cells were maintained in Eagle’s minimum essential medium, MCF-7, DLD-1 in RPMI 1640, SCC-25 in DMEM/F12 medium. To make the complete growth medium, fetal bovine serum (FBS) to a final concentration of 10% and 100 U/mL penicillin, 100lg/mL streptomycin were added. Cells were grown in a humidified incubator with 5% CO_2_ at 37 °C. The fresh culture medium was changed every 2–3 days. Cell culture media, FBS, trypsin, and antibiotics were used from Gibco (Thermo Fisher Scientific Inc., Waltham, MA, USA).

### 4.3. In Vitro Cytotoxicity of MTX and GLU–MTX

For MTT experiments, cell lines were seeded in 96-well plates (5−8 × 103 cells/well). The following day cells were treated with an appropriate complete culture medium (control) and different doses of methotrexate and glucose conjugated MTX (10, 50 µM) for 48 h.

After the incubation, an MTT assay was performed. Cell viability was evaluated by the conversion of the yellow tetrazolium salt (MTT) into violet formazan insoluble crystals in mitochondria of active cells. Following 4 h, the medium was removed, and the dye was dissolved by dimethyl sulfoxide (DMSO, Sigma-Aldrich, Munich Germany), creating the color, which intensity is proportional to the viable cells. The absorbance rate was measured at 490 nm, and the reference wavelength was 570 nm (Bio-TekBioTek ELX800 multi-well reader, BioTek, Winooski, VT, USA). The viable cells (VC) were calculated as VC (100%) = (absorbance of experimental group/absorbance of the control group) × 100%. MTT experiments were repeated, and figures represent the mean with standard deviation.

### 4.4. Measurement of Cellular Uptake of MTX and GLU–MTX by Mass Spectrometry

To measure differences in cellular uptake of Glu-Met and methotrexate, MCF-7 and SW480 cells were seeded in density 3 × 10^5^/well on 6 well plates. When cells reached 80% confluence, the medium was replaced with 1 mL/well fresh medium with or without tested compounds in dose 50 µM. Following 6 h of incubation, the medium was centrifuged, collected and stored immediately at −80 °C. Then adherent cells were washed once with 1 mL PBS at room temperature (RT). Next, the plate was placed on ice and washed with 500 ul ice-cold methanol: H2O (3:1) twice. The collected supernatant was centrifuged and stored at −80 °C until the analysis.

### 4.5. LC/MS Analysis

#### 4.5.1. Equipment

The UPLC system consisted of the Acquity UPLC binary pump, cooled sample manager and column oven (Waters, Milford, MA, USA). The mass spectrometer was a Xevo G2 Q-TOF MS equipped with an electrospray ionization interface (Waters, Milford, MA, USA). The data were acquired by using MassLynx software (version 4.0, Waters, Milford, MA, USA).

#### 4.5.2. LC Conditions

Chromatographic separation was performed using a Waters BEH Shield (1.7 µm, 2.1 × 100 mm) analytical column. The oven temperature was set at 45 °C. The mobile phases containing 0.1% formic acid in water (mobile phase A) and 0.1% formic acid in methanol (mobile phase B) were used at a flow rate of 0.2 mL/min. Gradient elution was performed according to the following steps: 1.0 min—5% B, 5.0 min—40% B, 7.5 min—65% B, 10 min—90% B, 11 min—90% B, 11.1 min—5% B, 13 min—5% B. The autosampler temperature was kept at 5 °C.

#### 4.5.3. MS Conditions

A mass spectrometer was interfaced with an electrospray ionization (ESI) probe. Mass spectra acquisition parameters were optimized using electrospray ionization (ESI) in the positive ionization mode. The temperatures were maintained at 120 °C and 450 °C for the source and desolvation line, respectively. The voltages were set at 0.5 kV and 40 V for the capillary and sampling cone, respectively. The desolvation gas and the cone gas (N2) flow rate was set at 800 L/h and 80 L/h, respectively.

### 4.6. Cell Cycle Analysis

After treatment, control cells, MTX and GLU–MTX-treated cells were harvested, collected and washed in PBS. Then, the cells were resuspended at 1−2 × 10^6^ cells/mL, and 5 mL of cold 70% ethanol was carefully added. Afterward, the cells were fixed for at least 1 h at 4 °C. Following washing twice in PBS, 0.5 mL of FxCycle™ PI/RNase staining solution (Life Technologies, Carlsbad, USA) to cell pellet was added and mixed well. The samples were incubated for 15–30 min at room temperature, protected from light and then analyzed by flow cytometry to determine the cell cycle profile.

### 4.7. Mouse Allograft Model of Human Breast Cancer

The animal use and care protocol were approved by the local ethical committee for animal experiments. The female BALB/c mice with a weight of 17–20 g (6–8 weeks old) were provided and maintained on free access to food and water. Then female BALB/c mice were injected subcutaneously with 4T1 breast cancer cell. The cells were suspended in 50 µL of Hanks’ solution: Matrigel (9:1) and implanted in the second right mammary gland (10^5^–10^6^ cells per mouse).

All animals were monitored for activity, physical condition, body weight, and tumor growth. Tumor size was determined every other day by caliper measurement of two perpendicular diameters of the implant. Tumor weight (in grams) was calculated by the formula *TV* = 1/2 × *a*2 × *b*, in which a is the long diameter, and b is the short diameter (in millimeters).

### 4.8. In Vivo Chemotherapy

The animals bearing breast cancer allograft tumors were randomly divided into two treatment groups and a control group (5–7 mice per group). Test animals received a single i.v. injection via the tail vein of GLU–MTX and MTX at a dose of 300 mg/kg, and 120 mg/kg, respectively. The treatment was started one day after the transplantation of tumor cells. The control animals received an injection of 0.2 mL of the vehicle only. The tumor volume and weight of each mouse were measured over a period of 18 days. The body weights of 4T1 tumor-bearing mice treated with GLU–MTX, MTX, and vehicle (DMSO) only were recorded simultaneously every 2 to 3 days during the study. No mice were lost during the experiment.

### 4.9. Histological Evaluation of Toxicity

Formalin-fixed and paraffin-embedded tissue sections of livers and lungs were stained with hematoxylin–eosin (HE) to evaluate the impact of the therapeutics on the tissues and 4T1 cells metastasis.

### 4.10. Statistical Analysis

In vivo data were analyzed by one-way analysis of variance (ANOVA). *p* < 0.05 was considered statistically significant. Data from in vitro experiments were expressed as means ± standard deviation (SD), and the statistical analysis was performed using Mann–Whitney U test in the PAST 4.03 program. The differences between groups were considered significant at *p* < 0.05.

## 5. Conclusions

In conclusion, we have synthesized a novel GLU–MTX conjugate and have shown that it has broad-spectrum anticancer activity. The compound preferentially accumulates in and annihilates malignant cells while showing reduced accumulation and low toxicity in normal fibroblasts. These results collectively represent a critical step forward in developing molecular tumor-targeting properties into established therapeutic drugs for improved safety and efficacy of anticancer therapies. These studies are essential for further preclinical and clinical development of a glucose-based class of compounds.

## 6. Patents

The authors are inventors on submitted patent applications (serial number P.426731).

## Figures and Tables

**Table 1:** Cell cycle progression data presented as mean ± standard deviation (SD).

	G0/G1	S	G2/M
Control	65.4 ± 2.1	22.99 ± 0.8	10.7 ± 0.6
MTX	23.05 ± 1.4	50.97 ± 2.3	17.17 ± 1.3
GLU–MTX	23.83 ± 1.7	50.61 ± 1.9	16.11 ± 1.8

## Data Availability

Data are contained within the article.

## References

[1] Khan Z.A., Tripathi R., Mishra B. (2012). Methotrexate: A detailed review on drug delivery and clinical aspects. Expert Opin. Drug Deliv..

[2] Howard S.C., McCormick J., Pui C., Buddington R.K., Harvey R.D. (2016). Preventing and Managing Toxicities of High-Dose Methotrexate. Oncologist.

[3] Świerkot J. (2007). Toxicity of low dose methotrexate in rheumatoid arthritis. Adv. Clin. Exp. Med..

[4] Abolmaali S.S., Tamaddon A.M., Dinarvand R. (2013). A review of therapeutic challenges and achievements of methotrexate delivery systems for treatment of cancer and rheumatoid arthritis. Cancer Chemother. Pharmacol..

[5] Mahato R., Tai W., Cheng K. (2011). Prodrugs for improving tumor targetability and efficiency. Adv. Drug Deliv. Rev..

[6] Liberti M.V., Locasale J.W. (2016). The Warburg Effect: How Does it Benefit Cancer Cells?. Trends Biochem. Sci..

[7] Calvaresi E.C., Hergenrother P.J. (2013). Glucose conjugation for the specific targeting and treatment of cancer. Chem. Sci..

[8] Makuch S., Wozniak M., Krawczyk M., Pastuch-Gawołek G., Szeja W., Agrawal S. (2020). Glycoconjugation as a promising treatment strategy for psoriasis. J. Pharmacol. Exp. Ther..

[9] Patra M., Awuah S.G., Lippard S.J. (2016). Chemical Approach to Positional Isomers of Glucose-Platinum Conjugates Reveals Specific Cancer Targeting through Glucose-Transporter-Mediated Uptake in Vitro and in Vivo. J. Am. Chem. Soc..

[10] Agrawal S., Wozniak M., Luc M., Walaszek K., Pielka E., Szeja W., Pastuch-Gawolek G., Gamian A., Ziolkowski P. (2017). Insulin and novel thioglycosides exert suppressive effect on human breast and colon carcinoma cells. Oncotarget.

[11] Ma Y., Wang W., Idowu M.O., Oh U., Wang X.Y., Temkin S.M., Fang X. (2019). Ovarian cancer relies on glucose transporter 1 to fuel glycolysis and growth: Anti-tumor activity of BAY-876. Cancers.

[12] Dyshlovoy S.A., Pelageev D.N., Hauschild J., Borisova K.L., Kaune M., Krisp C., Venz S., Sabutskii Y.E., Khmelevskaya E.A., Busenbender T. (2019). Successful targeting of thewarburg effect in prostate cancer by glucose-conjugated 1,4-naphthoquinones. Cancers.

[13] Barbosa A.M., Martel F. (2020). Targeting glucose transporters for breast cancer therapy: The effect of natural and synthetic compounds. Cancers.

[14] Tomaszowski K.-H., Hellmann N., Ponath V., Takatsu H., Shin H.-W., Kaina B. (2017). Uptake of glucose-conjugated MGMT inhibitors in cancer cells: Role of flippases and type IV P-type ATPases. Sci. Rep..

[15] Pohl J., Bertram B., Hilgard P., Nowrousian M.R., Stüben J., Wießler M. (1995). D-19575-a sugar-linked isophosphoramide mustard derivative exploiting transmembrane glucose transport. Cancer Chemother. Pharmacol..

[16] Woźniak M., Pastuch-Gawołek G., Makuch S., Wiśniewski J., Ziółkowski P., Szeja W., Krawczyk M., Agrawal S. (2021). Overcoming hypoxia-induced chemoresistance in cancer using a novel glycoconjugate of methotrexate. Pharmaceuticals.

[17] Patra M., Johnstone T.C., Suntharalingam K., Lippard S.J. (2016). A Potent Glucose-Platinum Conjugate Exploits Glucose Transporters and Preferentially Accumulates in Cancer Cells. Angew. Chem. Int. Ed..

[18] Young C.D., Lewis A.S., Rudolph M.C., Ruehle M.D., Jackman M.R., Yun U.J., Ilkun O., Pereira R., Abel E.D., Anderson S.M. (2011). Modulation of glucose transporter 1 (GLUT1) expression levels alters mouse mammary tumor cell growth in vitro and in vivo. PLoS ONE.

[19] Schwartz L., Supuran C., Alfarouk K. (2017). The Warburg Effect and the Hallmarks of Cancer. Anticancer Agents Med. Chem..

[20] Hanahan D., Weinberg R.A. (2011). Hallmarks of cancer: The next generation. Cell.

[21] Fu J., Yang J., Seeberger P.H., Yin J. (2020). Glycoconjugates for glucose transporter-mediated cancer-specific targeting and treatment. Carbohydr. Res..

[22] Deng D., Yan N. (2016). GLUT, SGLT, and SWEET: Structural and mechanistic investigations of the glucose transporters. Protein Sci..

[23] El Hilali M., Reux B., Debiton E., Leal F., Galmier M.J., Vivier M., Chezal J.M., Miot-Noirault E., Coudert P., Weber V. (2017). Linker structure-activity relationships in fluorodeoxyglucose chlorambucil conjugates for tumor-targeted chemotherapy. Bioorg. Med. Chem..

[24] Krawczyk M., Pastuch-Gawołek G., Pluta A., Erfurt K., Domiński A., Kurcok P. (2019). 8-hydroxyquinoline glycoconjugates: Modifications in the linker structure and their effect on the cytotoxicity of the obtained compounds. Molecules.

[25] Zemplén G., Pacsu E. (1929). Über die Verseifung acetylierter Zucker und verwandter Substanzen. Ber. Dtsch. Chem. Ges. (A B Ser.).

